# A Fifteen-year Review of Lymphomas in a Nigerian Tertiary Healthcare Centre

**DOI:** 10.3329/jhpn.v29i4.8446

**Published:** 2011-08

**Authors:** A. Olayiwola Oluwasola, John A. Olaniyi, Jesse A. Otegbayo, Gabriel O. Ogun, Titi S. Akingbola, Cornelius O. Ukah, Effiong E.U. Akang, Yetunde A. Aken'Ova

**Affiliations:** ^1^Departments of Pathology; ^2^Hematology,; ^3^Medicine, College of Medicine, University of Ibadan, Ibadan, Nigeria

**Keywords:** Acquired immunodeficiency syndrome, HIV, Lymphomas, Retrospective studies, Africa

## Abstract

In Africa, epidemiological data on the effect of the HIV epidemic on the occurrence of lymphomas are scanty. The 1990s witnessed the alarming rates of HIV/AIDS in Nigeria. The prevalence of HIV/AIDS in Nigeria increased from 1.8% in 1991 to 4.4% in 2005. The aim of this study was to determine whether there have been any changes in the frequency and pattern of lymphomas in view of the HIV/AIDS epidemic in the country. This is a retrospective study of all lymphoma cases diagnosed during 1991-2005. The prevalence of lymphomas declined from 1.4% to 0.7% of surgical biopsies during 1991-2005. There was a decline in the proportion of high-grade non-Hodgkin lymphoma and Burkitt's lymphoma from 79.1% and 45.8% respectively to 21.1% and 13.6% respectively. There is a suggestion that the HIV/AIDS epidemic in the country may not have influenced the pattern of occurrence of both major histomorphological types of lymphoma in Ibadan.

## INTRODUCTION

Lymphomas are a heterogeneous group of neoplasms of the lymphoid tissues traditionally categorized as either Hodgkin's lymphoma (HL) or non-Hodgkin's lymphoma (NHL), each displaying distinct behavioural, prognostic and epidemiological characteristics, with varying responses to treatment. Approximately 287,000 new cases of NHL are reported in the world each year (1). This disease affects more males than females, and the incidence increases with age. In most African populations, NHL is relatively rare but the relative frequency is above the world average in North and sub-Saharan Africa because of the high incidence of Burkitt's lymphoma (BL) in children in the tropical zone of Africa (2).

According to the 1976 report of the International Agency for Research on Cancer, Ibadan registered one of the highest incidences of lymphomas worldwide (3). The prevalence of BL, although still the commonest childhood malignancy in Ibadan, accounting for 19.4% of all childhood malignancies in the 1990s, declined from 50% in the 1960s to 37% in the 1970s and 1980s (4). This decline has been ascribed to improved living conditions, greater control of malaria, and probably decreasing infection due to Epstein Barr Virus (EBV) (4,5).

One of the most striking tumour associations in HIV-related disease is the development of high-grade B-cell lymphomas. The high increases reported in the incidence of NHL in many parts of the world, particularly in developed countries, have been attributed to the HIV pandemic (6,7). NHLs constitute an AIDS defining disease in 2-3% of patients (8).

In Africa, scanty epidemiological data are available on the effect of the HIV epidemic on the frequency of lymphomas and malignancies in general (9-11). The 1990s witnessed the alarming rates of HIV/AIDS in Nigeria. The prevalence of HIV/AIDS in Nigeria increased from 1.8% in 1991 to 5.8% in 2001 before eventually falling to 4.4% in 2005, with hot spot areas having rates as high as 21% (12). The effect of the HIV/AIDS epidemic on the pattern of lymphomas in Nigeria is yet to be defined. There has also not been any other comprehensive review of lymphomas in Ibadan since the last report in 1991 (13). We, therefore, decided to review all cases of lymphomas diagnosed during 1991-2005. The aim was to find out if any changes have been occurred in the frequency and pattern of lymphomas since the last review, which is a period particularly characterized by a rising HIV/AIDS epidemic in the country.

## MATERIALS AND METHODS

This is a retrospective study of all cases with histological or cytological diagnosis of lymphomas registered in the Department of Pathology, University College Hospital (UCH), Ibadan, Nigeria, over a 15-year period from January 1991 to December 2005.

The records of patients diagnosed to have lymphomas over the study period were obtained from the archival surgical and cytology registers of the department and from the Ibadan Cancer Registry, with a coverage population of about two million.

The NHL cases were categorized using the Working Formulation classification scheme while the HL cases were classified according to the Rye classification. The histological slides of cases not classified according to the Working Formulation in the original histology report were reviewed and reclassified. Where the original histological sections were inadequate or unavailable, the paraffin blocks were retrieved from the archives of the Department of Pathology, UCH, Ibadan, and fresh haematoxylin-eosin-stained sections were prepared and re-examined. Immunohistochemical examination was not performed on these cases as this service was not available in the institution during the study period. The HIV status of the patients was also not available since this test was not being routinely performed during the study period.

### Analysis of data

Data obtained were analyzed using the SPSS software (version 11). Student's *t*-test and Spearman's correlation test were applied where appropriate, with level of significance set at p≤0.05.

### Ethical aspects

This study was performed in compliance with the guidelines of the Helsinki Declaration on biomedical research on human subjects. It was a retrospective study, and confidentiality of the identity of the patients and personal health information was maintained.

## RESULTS

Six hundred and thirty-eight cases of lymphomas were recruited, comprising 421 (66%) histologically and 217 (34%) cytologically-diagnosed cases. These accounted for 0.9% of 47,177 surgical biopsies and 1.2% of 17,868 cytology specimens processed in the Department of Pathology during the study period.

**Fig. 1.**
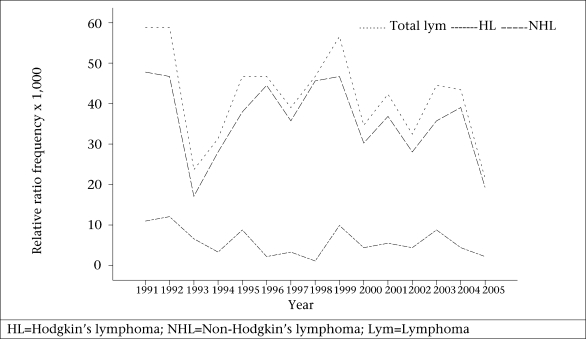
Relative ratio frequency and time trends for lymphomas

There were 558 (87.5%) cases of NHL and 80 (12.5%) cases of HL. There was a progressive decline in the annual frequency of lymphoma from 58 cases in 1991 to 24 cases in 2005 (Fig. 1), which represents a decrease from 1.4% to 0.7% of all surgical biopsies received during the same years in the Department of Pathology. The decrease was significant (χ^2^=17.4, p<0.0003). The decline was independently demonstrated in both NHL and HL, and the decline in these individual subtypes was significant (χ^2^=12.11, p=0.005 and χ^2^=6.01, p=0.014 respectively). It was also observed that the relative ratio frequency of malignant lymphoma among other registered cancers fell from about 4.1% in 1991 to about 1.6% in 2005.

Overall, there were 387 (60.7%) male patients and 251 (39.3%) female patients, representing a male:female ratio of 1.5:1. The age of patients with lymphoma ranged from one year to 85 years, with a mean age of 29.8 years. The second decade of life experienced the peak occurrence of lymphomas.

The nodal sites were slightly more often involved (53.6%) than the extranodal sites (46.4%). Most (98.7%) extranodal lymphomas were NHL, and the majority (52.1%) of the cases occurred in children aged less than 17 years.

### Non-Hodgkin's lymphoma

There were 336 (60.2%) male patients and 222 (39.8%) female patients with NHL, with a male: female ratio of 1.5:1. Their mean age was 29.6 years, and their ages ranged from one year to 85 years. Forty-eight percent of the cases occurred in the first two decades of life.

Two hundred and thirty-five (53.5%) cases were extranodal while 204 (46.5%) were nodal. The topography of 109 (21.3%) cases was not available. The commonest extranodal sites included the jaw (23.5%), nasopharynx and nasal cavity (7.4%), breast (5.2%), ovary (5.2%), and the gastrointestinal tract (4.9%). The commonest nodal sites of involvement were the cervical (38.5%), axillary (17.8%), and inguinal (9.4%) regions.

The commonest histological types of NHL were small non-cleaved cell (BL) (29.9%), diffuse large cell (15.5%), lymphoblastic (12.4%), and small lymphocytic lymphomas (9.6%) (Table 1). The jaw was the commonest site of occurrence of BL (37.1%), followed by the abdomen (16.8%), ovary (7.9%), and the breast (6.3%). The average age of occurrence of BL was lower in the jaw and abdomen (8.7 and 9.8 years respectively) compared to the ovaries and breast (20.3 and 15.9 years respectively).

**Fig. 2.**
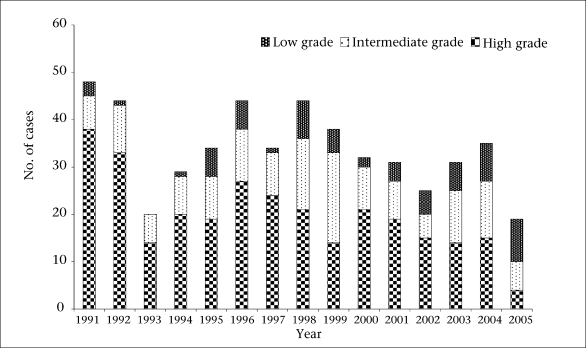
Trend in relative frequency of grades of non-Hodgkin lymphoma

Of 508 cases which had grading from histology and cytology, 298 (58.7%) were high-grade lymphoma, and 145 (28.5%) were of intermediate grade while 65 (12.8%) were low-grade lymphomas (Fig. 2). There was a significant decline in the comparative proportion of high-grade NHL compared to intermediate and low-grade NHL from about 79.1% in 1991 to about 21.1% in 2005 (χ^2^=19.65, p<0.0001). A similar decline was observed in the relative ratio frequency of BL from an average of 45.8% in 1991 to 15.8% in 2005. The difference was also significant (χ^2^=5.25, p<0.03).

**Table 1.** Age range, male:female ratio, relative frequency, and anatomic localization of lymphomas

Histological type	Age range (years)	Male: female ratio	Frequency		Anatomical localization nodal/extranodal
			No.	%	
Hodgkin's lymphoma					
Nodular sclerosis	11-74	1.4:1	32	5.0	31/0
Lymphocyte predominant	8-71	2:1	3	0.5	3/0
Mixed cellularity	5-56	2.4:1	17	2.7	15/1
Lymphocyte depleted	13-65	2:1	9	1.4	6/2
Others*	12-71	1.7:1	19	3.0	16/0
Total			80	12.5	71/3
Non-Hodgkin's lymphoma					
Small lymphocytic	11-81	1.4:1	61	9.6	36/21
Follicular predominantly small cell	-	-	1	0.2	1/0
Follicular mixed small and large cell	28-46	1:2	3	0.5	1/1
Follicular predominantly large cell	24-46	2:1	3	0.5	3/0
Diffuse small cleaved cell	11-67	1:1.3	9	1.4	4/3
Diffuse mixed small and large cell	8-78	2.4:1	34	5.3	16/13
Diffuse large cell	6-81	1.4:1	99	15.5	39/44
Large cell immunoblastic	6-85	2.1:1	28	4.4	21/3
Lymphoblastic	5-78	1.5:1	79	12.4	32/36
Small non-cleaved cell	1-39	1.3:1	191	29.9	17/117
Others*	4-80	2.5:1	50	7.8	34/7
Total			558	87.5	204/235

*Cases without histological subtypes

All the histological subtypes of NHL with sample-sizes of over 30 showed a male preponderance. The age of the patients diagnosed to have small non-cleaved cell lymphoma (BL) ranged from one year to 39 years (mean 10.6 years), and the peak age of occurrence was in the first decade of life. Most (92%) patients were aged 1-14 years.

### Hodgkin's lymphoma

HL was diagnosed in 51 (63.8%) male patients and in 29 (36.3%) female patients, giving a male:female ratio of about 1.8:1. The age range of occurrence was 5-74 years, with the mean age of 31.1 years. Sixty-six percent of the cases had HL before the age of 40 years while the modal age-group of occurrence was in the third decade of life. Most (88.8%) were nodal while 11.3% occurred at the extranodal sites, such as the intestine, liver, and spleen. The commonest nodal sites of involvement were the cervical, supraclavicular and axillary regions, in the descending order of frequency.

The commonest histological subtypes encountered were the nodular sclerosis (40%), followed by the mixed cellularity (21.3%) subtypes (Table 1). The lymphocyte-depleted subtype was most frequently diagnosed at the extranodal sites. All the histological subtypes showed a male preponderance.

The mean age and age range of the various histological subtypes of HL are shown in Table 2. The nodular sclerosis subtype was diagnosed in patients aged 11-74 years, with a mean age at diagnosis of 27 years. The age range of the lymphocyte-depleted subtype, however, was 13-65 years, with an average age of 43.1 years.

## DISCUSSION

It is estimated that approximately 100,000 new cases of cancer occurred yearly in Nigeria in the 1990s and that at the turn of the century, about 500,000 new cases will occur annually (14). Lymphomas were the commonest tumours observed in Ibadan, Nigeria, during 1960-1980 (15), and Ibadan Cancer Registry data from a study in 2000 revealed that the comparative frequency of lymphoma in Ibadan declined in the recent decades (16). A similar levelling off of previous rates of increase and decrease in incidence rates of NHL has been recently reported in some Nordic countries and in the USA, albeit for reasons different from ours (17,18).

Earlier reports of decline in the incidence of BL have been ascribed to improved living conditions and greater control of malaria probably decreasing infection due to EBV, reduced referral load in the University College Hospital, Ibadan, and improved cancer registration procedures (4,5). While these various factors may still have a role in the current trend in the occurrences of lymphomas, the extent of their specific contributions needs to be explored in future studies. To determine whether the trends are real, there is a need to keep in mind various artefactual factors that could influence the reported incidence without changing the true rate. These factors include completeness of registration, changes in disease classification, and advances in diagnostic technology (6). There have also not been any significant decrease in staff strength or infrastructure of this registry that could have affected the data-collection process. Similarly, changes in the international coding of disease in recent decades cannot account for significant losses of lymphoma cases. A more likely source of an artefactual effect might have resulted from advances in diagnostic technology; this, however, would have resulted in the detection of more cases over time, which is not our present experience. Nevertheless, this centre has only acquired a few additional diagnostic technologies in recent decades, mainly for radiological diagnosis.

**Table 2.** Age variables and male to female ratios of lymphoma patients

Histological type	Age range (years)	Mean age (years) (95% confidence intervals)	Male: female ratio
Hodgkin's lymphoma			
Nodular sclerosis	11-74	27 (21.5-32.5)	1.4:1
Lymphocyte predominant	8-71	35 (-1.2-71.9)	2:1
Mixed cellularity	5-56	27 (19.5-35.0)	2.4:1
Lymphocyte depleted	13-65	43 (32.0-54.3)	2:1
Others*	12-71	34 (25.8-44.1)	1.7:1
Non-Hodgkin's lymphoma			
Small lymphocytic	11-81	56 (51.6-60.2)	1.4:1
Follicular predominantly small cell	-	-	-
Follicular mixed small and large cell	28-46	34 (22.2-45.8)	1:2
Follicular predominantly large cell	24-46	32 (18.8-45.8)	2:1
Diffuse small cleaved cell	11-67	41 (28.2-54.0)	1:1.3
Diffuse mixed small and large cell	8-78	36 (29.5-43.0)	2.4:1
Diffuse large cell	6-81	41 (37.7-45.2)	1.4:1
Large cell immunoblastic	6-85	50 (41.7-57.9)	2.1:1
Lymphoblastic	5-78	22 (18.1-26.8)	1.5:1
Small non-cleaved cell	1-39	10 (9.8-11.3)	1.3:1
Others*	4-80	45 (38.6-50.4)	2.5:1

*Cases without histological subtype

HIV infection has been associated with a 60-fold increased risk of developing NHL in Western countries (19), and approximately 5-10% of HIV-infected persons will develop a lymphoma (20). The risk of HL is also increased in patients with HIV infection. In Africa however, the rate of association of NHL with HIV is 2.3-12.3% (21). The prevalence of HIV in Nigeria has consistently increased from 1.8% in 1991 to 5.8% in 2001 before a decline to 5% in 2003 and 4.4% in 2005 (12). Contrary to the experience in the United States and Europe (22), it is rather surprising to observe that this trend in the HIV epidemic (with an almost three-fold increase over the study period) in the country did not have any remarkably positive impact on the frequency of lymphoma in Ibadan during the study period. Suffice to mention at this point that the low frequency of lymphoma among our patients may be directly attributable to the fact that only a very small percentage of the estimated 3.9 million Nigerians with HIV infection receive the much-needed antiretroviral therapy while the majority succumbs to opportunistic infections. These two reasons may, therefore, severely limit the life expectancy in HIV patients before a lymphoma can develop or be diagnosed.

In this study while there was a preponderance of high-grade histological subtypes of NHL, their comparative proportion has drastically declined after an initial marginal increase from an average proportion of about 71.7% of all NHL cases reported in the series by Okpala *et al*. (13) during 1974-1989, from 79.1% in 1991 to about 18.2% in 2005. This decline involved the three histological subtypes of high-grade NHL, including BL. The prevalence of extranodal NHL has risen remarkably over the decades from a reported prevalence of 9.8% found by Thomas *et al*. during 1981-1998 (23) to 53.5% in the present study. However, this is still relatively low when compared with the prevalence rates of 76% (24) and 87% (25) reported in the series of HIV patients. There were no cases of intracerebral or primary body cavity NHL. These findings further corroborate the lack of impact of the HIV epidemic on the pattern of occurrence of lymphoma in the country since these two types of NHL are AIDS defining entities. The demographic patterns of various histological grades of NHL did not significantly differ from the last local review (13).

In Europe and the United States, NHL is the commonest malignancy in paediatric AIDS patients, and approximately one-third of cases are BL (22). There are three clinical subtypes of BL: endemic (eBL), sporadic (sBL), and AIDS-related BL (AIDS-BL). Characteristically, eBL occurs almost exclusively in Africa, has a peak of incidence at seven years of age, a preponderance of males over females, and is associated with EBV in virtually 100% of cases. The predominant sites of involvement are the jaws and abdomen (26). AIDS-BL occurs with a peak at 10-19 years of age and frequently involves lymph nodes and the bone marrow (27). In this study however, the modal age-group of occurrence of BL was in the first decade, jaw tumours were the commonest modes of presentation, and sites commonly associated with AIDS-BL, namely bone marrow and lymph nodes, were infrequently involved. There was no record of involvement of bone marrow while nodal manifestation represents 8.9% of all cases seen.

HIV-associated HL presents in an aggressive fashion, often with extranodal or bone marrow involvement (28). A distinctive feature of HIV-associated HL is the lower frequency of mediastinal lymphadenopathy compared to non-HIV-associated HL (29). In addition, most cases are of either mixed cellularity or lymphocyte-depleted subtype and show expression of EBV-associated proteins in Reed-Sternberg cells. In this series, HL accounted for 12.5% of all lymphoma cases over the study period. Extranodal involvement was infrequent having occurred in only about 11.3% of the patients. The most frequently-encountered histological subtype is the nodular sclerosis type, which was diagnosed in 40% of the 80 cases seen. It was followed by the mixed cellularity subtype. There were no cases of bone marrow or mediastinal involvement. It is, however, not surprising that the only cases of intestinal, splenic and liver involvement were of the mixed cellularity and lymphocyte-depleted histological subtypes. The latter two histological subtypes only constitute about 32.6% of all the cases. The relatively-infrequent occurrence at the extranodal sites and the rarity of lymphocyte-depleted subtype in this series lend credence to the inference that the HIV epidemic in the country has not significantly impacted on the pattern of occurrence of lymphoma seen here.

The significance of the finding of a lack of significant fingerprint of the HIV/AIDS epidemic on the epidemiology of lymphoma in this region of the country is that it corroborates earlier assertion that the degree of immune dysfunction at AIDS diagnosis, as measured by CD4 cell counts, was less in Africa than in industrial countries, and the median survival times are much shorter (30). As the risk of NHL in AIDS and other immunodeficiency states is related to the degree of immune dysregulation (31), it could be that the apparently low risk of HIV-associated lymphoma in Nigeria, as in some other African countries, is the result of competing mortality, particularly from infectious diseases in AIDS patients with relatively low levels of immunosuppression.

The unavailability of immunohistochemistry, flow cytometry, and cytogenetics precluded the re-categorization of these malignancies according to the recent World Health Organization (WHO)'s classification of haemato-lymphoid malignancies by cytogenetic studies for further sub-classification of our cases.

### Conclusions

While lymphomas still rank among the leading cancers in Ibadan, Nigeria, its incidence has, however, declined in recent decades. This decline was independently demonstrated in both NHL and HL. The HIV/AIDS epidemic being experienced in the country may not have influenced the pattern of occurrence of both major histomorphological types of lymphoma in Ibadan. However, our study was limited by our inability to use the WHO's classification of haemato-lymphoid malignancies and the non-availability of the HIV status of the patients. To this effect, a prospective study is being embarked on by this group involving serological analysis of HIV in lymphoma patients, and this hopes also to incorporate the WHO's classification of malignant lymphomas as immunohistochemistry service has commenced in our centre.
